# Efficient Production of High-Purity Magnesium Hydroxide from Serpentinite

**DOI:** 10.3390/molecules30173484

**Published:** 2025-08-25

**Authors:** Abdrazakh Auyeshov, Kazhmukhan Arynov, Chaizada Yeskibayeva, Aitkul Ibrayeva, Assel Zhumadildayeva

**Affiliations:** 1 Scientific Research Laboratory “Applied Chemistry”, M. Auezov South Kazakhstan University, Tauke-Khan St. 5, Shymkent 160012, Kazakhstan; 2 LLP “Institute of Innovative Research and Technology”, 8th Microdistrict, Building 28, Apt. 2, Almaty 050010, Kazakhstan

**Keywords:** magnesium hydroxide, magnesium sulfate, serpentinite, resource efficiency, energy efficiency

## Abstract

This article presents a technology for the production of magnesium hydroxide from serpentinite via sulfuric acid leaching of magnesium and purification of the resulting sulfate solution from impurity metals using thermally activated serpentinite (TA-SP) at 750 °C for one hour. Purifying the leach solution is one of the key challenges in obtaining high-purity magnesium compounds from serpentinite. It has been established that the use of thermally activated serpentinite to neutralize the acidic suspension of serpentinite to pH 8.3, prior to treatment with an alkaline agent (sodium hydroxide), has a positive effect on the purity of the precipitated magnesium hydroxide. The influence of the thermal treatment on the acid–base properties of serpentinite, its phase composition, and adsorbent structure parameters, such as specific surface area and micropore distribution, was studied, revealing improvements in the adsorption properties. Flowcharts for the acid leaching and magnesium hydroxide precipitation processes are provided. The flow-sheet that we propose is shown to reduce the number of steps in the process and amount of equipment required for the purification of sulfate solution while ensuring that the magnesium hydroxide product has a purity of at least 99.5%.

## 1. Introduction

The practical significance of magnesium rocks and their processing products is determined by their broad applicability across various industrial sectors. Among magnesium compounds, magnesium hydroxide is one of the most widely used and in-demand materials. It is utilized as a flame retardant in the production of thermoplastics and polymer composites, as a flocculant in the treatment of natural and industrial wastewater, and in the chemical and pharmaceutical industries, among others.

Currently, synthetic magnesium hydroxide is typically produced via a “wet” process, which involves the interaction of aqueous solutions of magnesium salts (MgCl_2_, MgSO_4_) with sodium hydroxide, followed by precipitation, filtration, washing, drying, and grinding of the product. In addition, surface modification of synthetic magnesium hydroxide may occur during the drying and grinding stages.

The quality of magnesium hydroxide obtained through the hydrochemical processing of enriched mineral raw materials is generally higher than that of the product derived directly from natural minerals, such as brucite or dolomite. This is attributed to the fact that hydrochemical precipitation processes allow for both solutions and suspensions to undergo purification stages. Serpentinite rocks are a promising source of magnesium due to their abundance; however, their industrial application remains limited owing to the absence of cost-effective processing technologies.

In recent decades, numerous technologies for serpentinite processing have been developed. Most of these technologies are based on chemical methods, including acid leaching (using sulfuric, hydrochloric, or nitric acid), sintering, and neutralization and purification techniques. Various studies have shown that high availability and magnesium extraction efficiency (85–90%) can be achieved with sulfuric acid [1]. Even higher selectivity has been demonstrated with hydrochloric acid, achieving yields exceeding 90% [2]. However, despite this advantage, the resulting magnesium nitrate solution is difficult to utilize and is associated with high processing costs [3]. The preliminary thermal activation of serpentinite [4] has been shown to improve the leaching efficiency by 20–30% when used in acid-based methods.

A considerable number of studies have focused on the decarbonization of serpentinite using various leaching agents [5,6,7]. However, a practical application for these proposals has not yet been found due to technological, economic, or environmental limitations.

The purification stage of magnesium-containing process solutions is one of the most critical steps in technologies based on the acid processing of serpentinite [8,9]. For example, to obtain high-purity Mg(OH)_2_, effectively purifying the pregnant solution for the material prior to precipitation is essential.

During acid leaching, in addition to Mg^2+^, undesirable impurities such as Fe^3+^, Al^3+^, Ca^2+^, and colloidal silica (SiO_2_·H_2_O) also enter the solution. The first three are typically co-precipitated as hydroxides (e.g., Mg(OH)_2_), while colloidal silica complicates the filtration and reduces the yield and purity of magnesium sulfate and subsequently magnesium hydroxide. Therefore, the purification of magnesium-containing solutions plays a crucial role in the process and significantly affects their economic efficiency.

In most studies focused on the neutralization and purification of process solutions, stepwise precipitation methods for the hydroxides of impurity metals (Fe^3+^, Al^3+^, Ni^2+^, etc.) have been proposed by increasing the pH (e.g., to 5–6). For example, [6] discusses the influence of various conditions (including pH) on solubility, which is important for the precipitation of Mg(OH)_2_ and for preliminary neutralization. Reagents such as NaOH, Na_2_CO_3_, Ca(OH)_2_, and MgO are used. Each of these methods has characteristic disadvantages, including an increased number of process steps, magnesium co-precipitation losses, and a limited availability of MgO, which negatively affect the technological and economic indicators.

Recent studies [3,8] have shown that the H_2_SO_4_-leaching → purification of thermally activated serpentinite → NaOH neutralization scheme enables the production of Mg(OH)_2_ with a purity of up to 97% and improved properties. Additionally, the process solution is purified more efficiently due to acid–base interactions between thermally activated serpentinite (TA-SP) and the acidic suspension medium.

In [9], a high sorption purification efficiency was demonstrated when using thermally activated serpentinite. It was shown that mechanochemically modified serpentinite exhibits a high sorption capacity for heavy metal ions, which enhances the efficiency of the purification of the solution during the Mg(OH)_2_ precipitation. This finding, as well as others, formed the basis for the ion-exchange-based process proposed in this study for the production of magnesium hydroxide from serpentinite.

In Kazakhstan, serpentinite (Zhitikarinskoe deposit) is considered to be a promising alternative source of magnesium for the production of its compounds [10], which emphasizes the importance of resource conservation within the framework of processing technogenic serpentinite waste. High-quality dolomite deposits have not been identified in Kazakhstan. The Zhitikarinskoe deposit has been operating for over 65 years, and this has resulted in the accumulation of large amounts of tailings (hundreds of millions of tons) from chrysotile ore processing.

Considering the above, our aim with this study is to investigate the technological processes that can be used to obtain magnesium hydroxide, including the use of thermally activated serpentinite for the neutralization and purification of the acidic leach solution prior to the precipitation of the target magnesium hydroxide, as well as to assess how these stages influence the quality of the final product.

This study integrates the green chemistry approach, efficient utilization of mineral resources, and production of high-purity inorganic compounds.

## 2. Results and Discussion

Leaching of PTW with sulfuric acid and production of MgSO_4_.The proposed technology for producing magnesium hydroxide from serpentinite comprises the following key chemical reactions:

Mg_3_Si_2_O_5_(OH)_4_ + 6H_2_SO_4_ → 3Mg(HSO_4_)_2_ + 2SiO_2_ + 5H_2_O (1)

Mg(HSO_4_)_2_ + 4NaOH → Mg(OH)_2_↓ + 2Na_2_SO_4_ + 2H_2_O (2)

Mg_3_Si_2_O_5_(OH)_4_ + 3H_2_SO_4_ → 3MgSO_4_ + 2SiO_2_ + 5H_2_O (3)

Mg^2+^ + H_2_SO_4_ → MgSO_4_ + 2H^+^ (4)

1.000 mol:1.046 mol:1.000 mol

In this molar ratio, 1.000 mol corresponds to the amount of magnesium ions (Mg^2+^) obtained from the PTW; 1.046 mol corresponds to the amount of sulfuric acid (H_2_SO_4_) added with a slight excess to compensate for the impurities present in the PTW; and 1.000 mol corresponds to the amount of magnesium sulfate (MgSO_4_) formed according to the reaction stoichiometry.

The process includes the following stages: the leaching of serpentinite material with sulfuric acid; the subsequent neutralization and purification of the resulting sulfate solution using thermally activated serpentinite (TA-SP) at 750 °C for 1 h; and finally, the precipitation of magnesium hydroxide from the purified magnesium sulfate solution using sodium hydroxide (NaOH) as the precipitating agent. The reaction mixture of PTW and sulfuric acid was prepared according to the proposed chemical Equation (3). Based on this, the molar ratio of the reagents used in the experiment was selected as shown in Equation (4).

The leaching process of the PTW with sulfuric acid was carried out in a three-neck round-bottom flask equipped with a thermometer, reflux condenser, sampling port, and mechanical stirrer.

Into the reaction flask, 500 g of PTW and 1000 cm^3^ of water were loaded. While stirring (200–300 rpm), 415 cm^3^ of 92% H_2_SO_4_ (density 1.824 g/cm^3^) was added in portions (150–200 cm^3^ each) from a dropping funnel to the suspension (PTW + H_2_O). The total weight of the reaction mixture was 2256 g, with a liquid-to-solid ratio of 4.5:1. The stoichiometric amount of H_2_SO_4_ (SNK) calculated based on the Mg^2+^ content in the PTW was 1.046:1.

Upon the addition of acid, the mixture heated up, and within 2–3 min, the temperature reached 100–105 °C, which significantly intensified the bubbling and suspension boiling. Therefore, the interval between the additions of acid was reduced to ensure moderate effervescence.

As a result, a thick, bluish-gray-colored suspension was formed. The measured pH value was 0.57. The leaching process was conducted over 3 h. Samples (10 mL each) were taken at 30, 60, 120, and 180 min for the chemical analysis. The suspension was filtered, the precipitates were rinsed with a small amount of water, and then the filter and solid residue were dried at 105 °C to constant weight and subjected to elemental analysis. The magnesium content was additionally monitored using an inductively coupled plasma mass spectrometer VARIAN 820-MS (Varian Inc., Mulgrave, Australia, 2007).

The analytical results are presented in Table 1.

An analysis of Table 1 shows that just 30 min after the start of the leaching process, the majority of the elements contained in the PTW had already diffused into the sulfate solution. Calcium began to appear in the solution after only 60 min. No significant changes in concentration were observed in the time interval between 30 and 180 min: the magnesium content in the solution increased by 12.8%, the iron content increased by 17%, while the silicon content decreased by 5.8%. In the insoluble residue, the magnesium content decreased by 27%, the silicon content increased by 5.5%, and the iron content decreased by 17%.

Thus, the primary acid–base interaction between PTW and H_2_SO_4_ occurs within the first 30 min. During this period, the amount of extractable magnesium sulfate from 100 g of PTW reached 168.65 g (≈70% of the theoretical yield). One of our key observations is that the mass of magnesium sulfate extracted between 30 and 180 min remained practically unchanged. The obtained values were 168.65, 168.90, 171.60, and 167.68 g, which indicate that there is no need to extend the process duration.

Neutralization and purification of the productive sulfate solution using thermally activated PTW (TA-PTW) at t = 750 °C. The next experiment was conducted under the same conditions as the first one. However, considering the results outlined in Table 2 and the slight increase in the amount of extractable magnesium sulfate between 30 and 180 min, the leaching time was limited to 30 min. After this period, 1.0 dm^3^ of distilled water was added to the acidic suspension. Thermally activated PTW (TA-PTW) was used as the neutralizing agent.

Effect of thermal treatment on the structure of PTW. The use of TA-PTW is justified by the fact that thermal activation leads to significant structural changes, including the increased porosity and enhanced reactivity of serpentinite [11]. Dehydroxylation occurs, resulting in the formation of an amorphous phase of silica and magnesium oxide according to the following reaction:

Mg_3_Si_2_O_5_(OH)_4_ → 3MgO + 2SiO_2(amorphous)_ + 2H_2_O (5)

**Figure 1:**
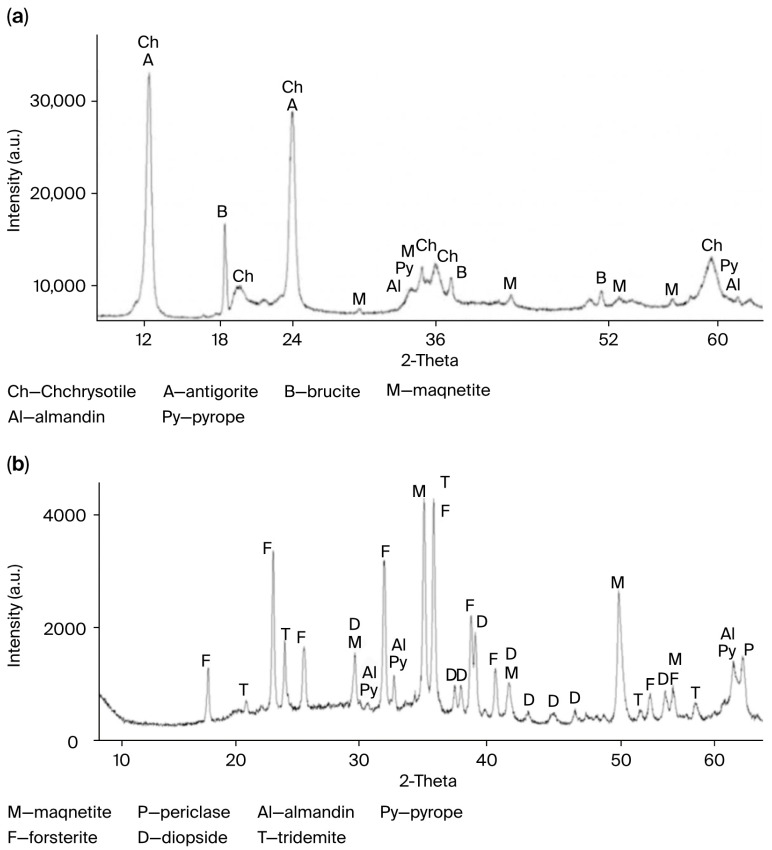
X-ray diffraction patterns: (**a**) initial powdery process tailing waste (PTW); (**b**) thermally activated PTW (TA-PTW) after treatment at 750 °C. Phase designations: M—magnetite; F—forsterite; D—diopside; T—tridymite; Py—pyrope; Al—almandine; P—periclase.

The qualitative change in the phase composition of the original PTW upon thermal treatment at 750 °C is shown in Figure 1a,b.

The activation of serpentinite during the thermal treatment occurs as a result of the internal rearrangement of the serpentinite crystal lattice, leading to the formation of periclase (MgO) (Figure 1b). This component enhances the alkaline properties of the material [12] and increases the number of active sites on the surface of TA-PTW particles.

Figure 1a shows that the XRD pattern of TA-PTW prior to the thermal activation exhibits sharp and intense peaks corresponding to chrysotile (Ch) and impurities such as antigorite (A), brucite (B), magnetite (M), almandine (Al), and pyrope (Py). After the thermal activation at 750 °C (Figure 1b), a significant decrease in the intensity of the main chrysotile peaks, peak broadening, and the appearance of new crystalline phases—periclase (P), forsterite (F), diopside (D), and tridymite (T)—are observed. These transformations indicate the dehydroxylation and amorphization of the mineral structure. Such changes confirm the destruction of the original crystalline lattice and the formation of intermediate thermal products, which, in turn, enhance the efficiency of the acid leaching.

The optimal conditions for the Mg(OH)_2_ synthesis process were established based on variations in the technological parameters, including the activation temperature of serpentinite, the amount of acid used for the activation, the pH of the neutralization medium, and the precipitation time. Based on the obtained data, process parameters ensuring high purity and yield of Mg(OH)_2_ were selected.

**Figure 2:**
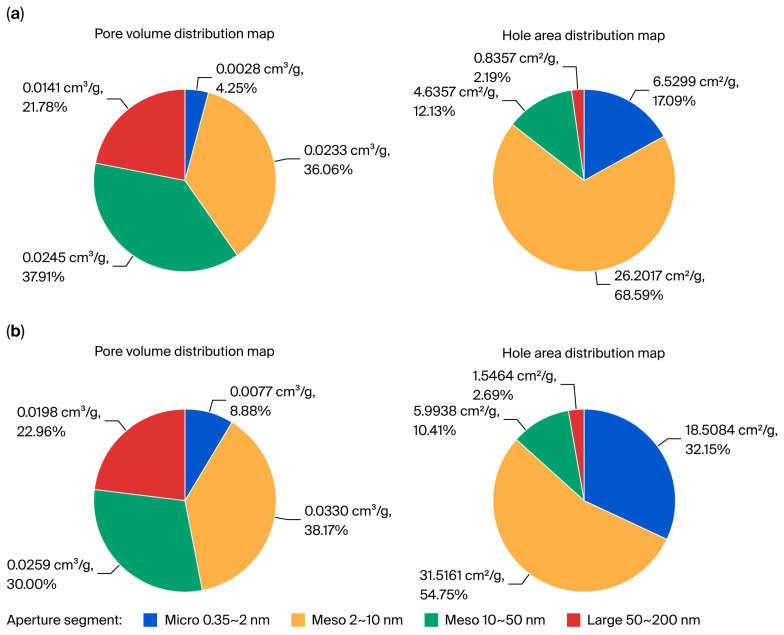
Pore volume and surface area distribution in (**a**) initial and (**b**) thermally activated PTW.

Additional studies on the effect of the thermal treatment on the specific surface area of the PTW (Figure 2) revealed a significant increase in the proportion of micropores (0.35–2.0 nm) in the thermally activated sample (b) compared with the initial PTW (a).

This confirms that TA-PTW exhibits a high adsorption capacity for Fe^3+^, Al^3+^, and Ca^2+^ ions due to its increased surface area and the number of active sites [9].

Neutralization process. The acidic suspension with TA-PTW was neutralized until a pH of 8.3 was reached. The resulting suspension was filtered, and the solid residue was washed twice with 0.5 L of distilled water.

The analytical results for the filtrate and residue after neutralization using TA-PTW are presented in Table 2.

Analysis of results. At this acidity (pH = 8.3), the filtration and washing of the suspension proceeded without difficulty. As shown in Table 3, the filtrate after neutralization is practically free of Fe^3+^, Al^3+^, and Si^4+^ ions, except for a negligible amount of Ca^2+^.

The magnesium sulfate yield after neutralization was 46% of the total magnesium content in PTW and TA-PTW. At the same time, the magnesium recovery from sulfuric acid reaches 98–100%. The MgSO_4_ concentration in the first filtrate was 243 g/L (24.3%), and in the repeated leaching of a new batch of PTW (with adjusted acidity), the MgSO_4_ concentration was 47–48%.

The residue (OS) after the neutralization of TA-PTW contained the following elements, wt%:

Mg = 11.91; Al = 0.56; Si = 23.60; S = 0.43; Ca = 0.30; Fe = 5.30.

The amorphous silica (SiO_2_·nH_2_O) in the insoluble residues can be used in various industries; for example, as an active mineral additive (microsilica) for high-strength concrete [8,9]; as a geopolymer precursor in alkaline activation [3]; as a soil improver for the reclamation of saline soils; and for pH [2] structure adjustment.

Identified advantages of using thermally activated PTW as a neutralizing agent. The application of thermally activated process tailing waste (TA-PTW) during the neutralization stage offers several advantages over the conventional reagent (NaOH):

1. It enhances the magnesium content in the productive solution due to acid–base interactions between TA-PTW and the acidic medium;
2. It does not introduce extraneous ions into the sulfate solution, unlike alkaline reagents;
3. Owing to its structural and adsorptive properties, TA-PTW enables the more effective removal of impurity metal ions (Fe^3+^, Al^3+^, etc.), with the exception of minor amounts of Ca^2+^.

Therefore, the use of TA-PTW provides a high-purity initial magnesium sulfate solution, suitable for the subsequent precipitation of magnesium hydroxide.

Precipitation of magnesium hydroxide (Mg(OH)_2_). Magnesium hydroxide was precipitated from the purified sulfate solution (MgSO_4_ concentration: 19–24%). A 25% sodium hydroxide solution was used as the precipitating agent (reagent grade, in accordance with GOST 4328-77).

The precipitation reaction proceeded according to the following equation:

Mg^2+^ + 2OH^−^ → Mg(OH)_2_↓ (6)

The reagent ratio was determined based on the following stoichiometric calculation:

Mg^2+^ (1 mol) + 2NaOH (1.05 mol) = Mg(OH)_2_ (1 mol) + 2Na^+^ (7)

To 250 mL of a 19.5% MgSO_4_ solution, 82 mL of a 25% NaOH solution was gradually added under stirring. During the reaction, the temperature of the mixture increased to 50 °C. At pH = 9.5, turbidity formation began intensively. The precipitation was considered to be complete at pH = 12.5. The suspension was stirred for 1 h at a low speed and then allowed to settle for decantation and cooling to take place.

After 2 h, the precipitate was filtered and washed via a decantation process, following this sequence: twice with a 0.01% NaOH solution and distilled water. The yield of magnesium hydroxide after drying at 105 °C to constant weight was 26.22 g (98%). Precipitation began at pH = 9.4 and was completed at pH = 12.4. The residual concentration of Mg^2+^ in the solution after complete precipitation was 10^−5^ mol/L.

**Figure 3:**
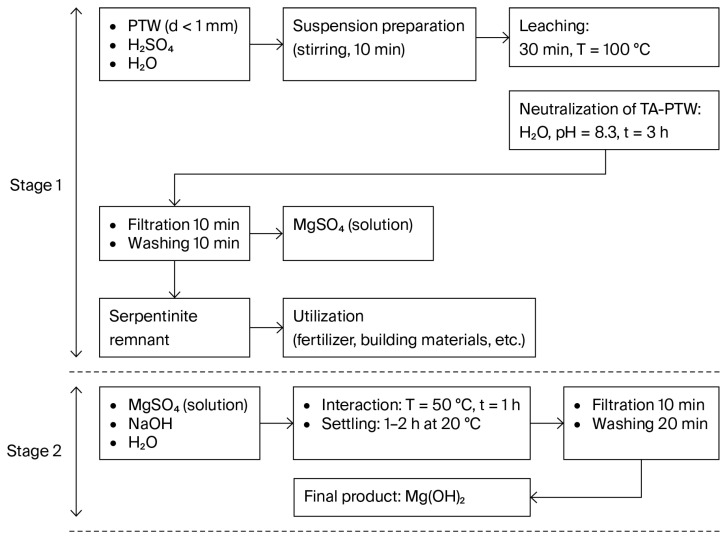
Process flow diagram for obtaining Mg(OH)_2_ from MgSO_4_ solution produced via the sulfuric acid leaching of serpentinite using thermally activated PTW. The scheme includes two stages: (1) leaching and neutralization of the suspension to obtain a purified MgSO_4_ solution; (2) precipitation of Mg(OH)_2_ using NaOH, followed by filtration and washing.

Figure 3 displays the process flow diagram for obtaining Mg(OH)_2_ from the MgSO_4_ solution (filtrate of sulfuric acid leaching) with a concentration of C(MgSO_4_) = 19–24% and outlines the process parameters (precipitation, washing, and drying).

The resulting Mg(OH)_2_ obtained according to the presented flow-sheet (Figure 3) contains the following (wt.%): Mg—99.5881; Al—0.0180; Na—0.0508; and Ca—0.2251.

From the results of our chemical analysis, we indicate that the use of thermally activated PTW (TA-PTW) during the neutralization and purification of the productive sulfate leach solution has a positive effect on the quality characteristics of the resulting magnesium hydroxide. This approach simplifies the technological process and may reduce the number of processing stages and equipment required for removing impurity metal ions from acidic leachates. Overall, it facilitates the production of high-purity magnesium hydroxide from serpentinite.

Economic aspects. A preliminary economic assessment of the proposed process was carried out. According to laboratory tests, the estimated resource and energy efficiency of producing 1 ton of Mg(OH)_2_ is approximately USD 495.0. The use of serpentinite waste and the absence of expensive reagents ensure that the technology is cost-effective. This assessment only considers the main stages of the process and does not include capital costs, which is typical of the preliminary technoeconomic justification at the laboratory stage.

Environmental aspects. The proposed technology offers several environmental advantages. First, it is based on the processing of technogenic waste (serpentinite tailings), meaning that waste is utilized rather than left to accumulate. Second, it does not require the use of expensive or toxic reagents. Third, the neutralized solutions formed during the neutralization and precipitation stages can be returned to the process or subjected to further treatment, thereby reducing the load on wastewater systems. Thus, this process can be considered an environmentally oriented approach to the utilization of mineral waste and the production of valuable magnesium compounds.

## 3. Materials and Methods

A powdered technogenic waste (PTW) obtained via chrysotile ore processing of the Zhitikarinskoe deposit (AO “Kostanay Minerals”, Kazakhstan) was used as the serpentinite-based material for this study. The PTW is a bluish-gray-colored fibrous powder consisting of fine solid particles free of lumps and large inclusions. It is generated and accumulated in dry dust collection systems during the crushing and fractionation of chrysotile raw material. The elemental composition of the PTW is shown in Table 3.

Thermal treatment of the PTW was carried out in a muffle furnace. The temperature of 750 °C and duration of 1 h for the thermal activation of serpentinite were selected based on the preliminary experiments, where we aimed to optimize the conditions for the dehydroxylation and amorphization of the mineral structure. It was established that such conditions facilitate the efficient removal of bound water and increase the solubility of magnesium in the subsequent stages of acid leaching. It was found that thermal activation contributes to a reduction in particle size, with no fraction larger than 0.9 mm remaining. The predominant fraction (94%) consists of particles within the range of 0.14–0.07 mm. The thermally treated material was additionally ground before use. The thermally activated PTW is hereinafter referred to as TA-PTW.

All the analytical procedures aimed to determine the distribution of the elements during leaching and solution purification using a JSM-6490LV scanning electron microscope (JEOL Ltd., Tokyo, Japan) equipped with an INCA Energy 350 energy-dispersive X-ray spectroscopy (EDS) system. Chemical analysis of the MgSO_4_ solution and the final Mg(OH)_2_ product was performed using an inductively coupled plasma mass spectrometer VARIAN 820-MS (Varian Inc., Mulgrave, Australia, 2007).

X-ray diffraction (XRD) patterns of the PTW and thermally activated PTW (TA-PTW) were obtained using a D8 Advance diffractometer (Bruker AXS GmbH, Karlsruhe, Germany), with Cu-Kα radiation at 40 kV and 40 mA. The resulting diffractograms were processed, and the interplanar spacings were calculated using EVA software (version 4.2). Phase identification was carried out using the Search/Match algorithm with the PDF-2 (JCPDS) powder diffraction database.

The specific surface area and pore size distribution were determined using the following principle: static capacity method using a BSD-660S A3 instrument (BSD Instrument Technology, Beijing, China).

The methodologies used for the acid leaching and Mg(OH)_2_ precipitation process are provided in the Results and Discussion Section to ensure clarity and continuity of presentation in this paper.

## 4. Conclusions

By performing the processes of leaching PTW with sulfuric acid and the neutralization and purification of the resulting sulfate solution using thermally activated PTW (TA-PTW), it is possible to obtain magnesium sulfate solutions with a high purity and favorable characteristics, thereby facilitating the production of high-purity magnesium hydroxide.

Thermally activated PTW demonstrated higher basicity and enhanced adsorption activity toward impurity metal ions. Its application during the neutralization and purification stages of the productive solution contributed to obtaining high-purity magnesium hydroxide. TA-PTW’s application is also associated with improved textural properties, increased specific surface area, and a greater proportion of micropores in its structure.

## Figures and Tables

**Table 1:** Elemental composition of the filtrate and insoluble residue during sulfuric acid leaching of PTW at 100–105 °C.

Element	Duration, min							
	30	60	120	180	30	60	120	180
	Filtrate, wt.%				Insoluble Residue, wt.%			
C	–	–	–	–	4.85	4.36	5.19	4.43
O	5.09	57.10	53.65	57.41	54.17	53.53	53.79	53.98
Mg	10.36	10.71	11.11	11.69	3.31	2.99	2.42	2.39
Al	0.48	0.42	0.37	0.30	0.33	–	–	–
Si	4.10	3.82	4.25	3.86	34.53	36.06	36.36	36.45
S	28.99	28.02	28.46	28.42	1.50	1.31	1.19	1.56
Ca	–	0.27	–	–	0.22	0.28	–	–
Fe	1.98	2.66	2.16	2.32	1.10	1.46	1.06	1.19
Total	100.00	100.00	100.00	100.00	100.00	100.00	100.00	100.00
m, g MgSO_4_	168.65	168.90	171.60	167.68	–	–	–	–

Note. The MgSO_4_ content was calculated based on the magnesium concentration in the filtrate, assuming the complete conversion of dissolved magnesium to magnesium sulfate.

**Table 2:** Elemental composition of the neutralized sulfate suspension (pH = 8.3), wt.%.

Element	Initial Substances		Neutralized Suspension		
	PTW	TA-PTW	Filtrate-1	Filtrate-2	Residue (OS)
C	2.58	2.54	–	–	9.35
O	51.00	45.22	59.90	61.90	48.55
Mg	25.00	27.89	16.60	15.64	11.91
Al	0.54	0.46	–	–	0.56
Si	17.45	19.17	–	–	23.60
S	–	–	23.50	22.21	0.43
Ca	0.50	0.53	–	0.25	0.30
Fe	2.93	4.19	–	–	5.30
Total	100.00	100.00	100.00	100.00	100.00

**Table 3:** Elemental composition of the PTW (wt.%).

**Element**	C	O	Mg	Al	Si	S	Ca	Fe	Total
**Content**	2.58	51.00	25.00	0.54	17.45	–	0.50	2.93	100.00

## Data Availability

The datasets generated and/or analyzed during the current study are available from the corresponding author upon reasonable request.

## Abbreviations

The following abbreviations are used in this manuscript:

SP	Serpentinite
TA-SP	Thermally activated serpentinite
PTW	Powdered technogenic waste
TA-PTW	Thermally activated PTW
SNK	Stoichiometrically necessary amount (of acid)
EDS	Energy-dispersive X-ray spectroscopy
XRD	X-ray diffraction
M	Magnetite
F	Forsterite
D	Diopside
T	Tridymite
Py	Pyrope
Al	Almandine
P	Periclase
